# Interpenetrating Polymer Networks of Poly(2-hydroxyethyl methacrylate) and Poly(N, N-dimethylacrylamide) as Potential Systems for Dermal Delivery of Dexamethasone Phosphate

**DOI:** 10.3390/pharmaceutics15092328

**Published:** 2023-09-15

**Authors:** Marin Simeonov, Bistra Kostova, Elena Vassileva

**Affiliations:** 1 Laboratory on Structure and Properties of Polymers, Faculty of Chemistry and Pharmacy, University of Sofia, 1, J. Bourchier blvd., 1164 Sofia, Bulgaria; ohtev@chem.uni-sofia.bg; 2 Department of Pharmaceutical Technology and Biopharmaceutics, Faculty of Pharmacy, Medical University of Sofia, 2, Dunav str., 1000 Sofia, Bulgaria

**Keywords:** interpenetrating polymer networks, hydrogels, dexamethasone sodium phosphate, drug delivery, dermal application

## Abstract

In this study, a series of novel poly(2-hydroxyethyl methacrylate) (PHEMA)/poly(N,N′-dimethylacrylamide) (PDMAM) interpenetrating polymer networks (IPNs) were synthesized and studied as potential drug delivery systems of dexamethasone sodium phosphate (DXP) for dermal application. The IPN composition allows for control over its swelling ability as the incorporation of the highly hydrophilic PDMAM increases more than twice the IPN swelling ratio as compared to the PHEMA single networks, namely from ~0.5 to ~1.1. The increased swelling ratio of the IPNs results in an increased entrapment efficiency up to ~30% as well as an increased drug loading capacity of DXP up to 4.5%. X-ray diffraction (XRD) and differential scanning calorimetry (DSC) show the formation of a solid dispersion between the drug DXP and the polymer (IPNs) matrix. Energy-dispersive X-ray (EDX) spectroscopy shows an even distribution of DXP within the IPN structure. The DXP release follows Fickian diffusion with ~70% of DXP released in 24 h. This study demonstrates the potential of the newly developed IPNs for the dermal delivery of DXP.

## 1. Introduction

Interpenetrating polymer networks (IPNs) are defined as two or more networks that are at least partially interlaced at the molecular scale but not covalently bonded to each other and cannot be separated unless chemical bonds are broken [1]. Many IPNs’ properties are advantageous for their application to drug delivery, such as controllable swelling degree, network density, and chemical composition. These properties strongly depend on the ratio between the IPN constituents, i.e., the drug release behavior from the IPNs can be controlled by varying the IPNs’ composition [2].

Dexamethasone sodium phosphate (DXP) is a glucocorticoid with a dose-dependent anti-inflammatory, anti-allergic, and immunosuppressive effect [3], which is applied for treating different skin conditions such as atopic dermatitis [4,5]. When applied as an injectable solution, DXP exhibits many side-effects including increased blood pressure, mood and behavior changes, high blood sugar, etc. [6]. Dermal administration of DXP allows for avoiding these complications as well as ensuring DXP local delivery right to the affected skin. 

PHEMA is a hydrophilic, biocompatible, and non-toxic polymer [7,8] that was developed and studied as a material for soft contact lenses [9] as well as for ophthalmic drug delivery [10]. So far, only a few studies have explored the potential of PHEMA as drug delivery systems for DXP, all of them being focused on DXP ocular delivery [11,12]. As PHEMA is a neutral polymer, the DXP loading in PHEMA hydrogels is enhanced by adding different positively charged components. For example, cationic surfactants, such as cetalkonium chloride [11], or cationic comonomers, such as 2-(diisopropylamino) ethyl methacrylate [12], are incorporated in the PHEMA networks to ensure electrostatic interactions between the anionic-DXP- and PHEMA-based delivery system. However, this approach is not always successful, as cationic constituents are known to be irritating to the skin [13] and their involvement is not always suitable. On the other hand, the incorporation of strongly hydrophilic components, such as PDMAM, known also for its biocompatibility [14] could be very beneficial for developing PHEMA-based DXP delivery vehicles. Moreover, using the IPN approach to combine PHEMA and PDMAM provides the opportunity to control the hydrophilicity as well as the swelling ability of the resulting material, thus also controlling the drug release properties of the material. To our knowledge, only one recent paper explores the potential of IPNs for the dermal delivery of DXP [15]. This is a multicomponent IPN based on a copolymer of PDMAM and poly(ethylene glycol) and poly(ethylene glycol) diacrylate crosslinked poly(N-isopropylacrylamide), which was successfully employed for DXP dermal delivery. 

This study is focused on the development of novel poly(2-hydroxyethyl methacrylate) (PHEMA)/poly(N,N′-dimethylacrylamide) (PDMAM) IPNs and on the exploration of their potential as dermal drug delivery systems of DXP. For this purpose, a series of IPNs with different PHEMA/PDMAM ratios were synthesized and characterized in terms of their swelling dependence of ionic strength, pH, and temperature. The loading capacity of the IPNs was studied using different DXP concentrations and the obtained DXP-PHEMA/PDMAM IPNs were characterized using X-ray diffraction (XRD), temperature-modulated differential scanning calorimetry (TMDSC), and attenuated total reflectance–Fourier transform infrared spectroscopy (ATR-FTIR) to study the DXP–polymer interaction. The DXP release profiles from the IPNs were studied under conditions simulating dermal administration.

## 2. Materials and Methods

### 2.1. Materials

2-Hydroxyethyl methacrylate (≥99%) (HEMA), N,N-dimethylacrylamide (99%) (DMAM), poly(ethylene glycol) diacrylate (average Mn 575) (PEGDA), 1-hydroxycyclohexyl phenyl ketone (99%) (HCHPK), citric acid, and sodium citrate monobasic (purum p.a., anhydrous, ≥99.0%) were purchased from Sigma-Aldrich (St. Louis, MO, USA). Potassium chloride (KCl), sodium chloride (NaCl), and sodium phosphate dibasic (Na_2_HPO_4_) were purchased by Fluka, Germany. All reagents were used as received, without further purification. Dexamethasone sodium phosphate (DXP) was purchased from Crystal Pharma (Valladolid, Spain). The chemical formulas and role of the reagents used within this study are shown in Table S1 (Supplementary Information).

### 2.2. IPN Synthesis

The PHEMA/PDMAM IPNs were synthesized following two-step sequential method. 

In the first step, PHEMA single networks (SNs) were prepared via bulk polymerization of HEMA monomer, containing 1 mol% PEGDA as crosslinking agent and 0.1 mol% HCHPK as UV-photo initiator. After the complete dissolution of the components, the obtained solution was placed in polystyrene mold under a UV-lamp (Analytik Jena GmbH, Jena, Germany) at 365 nm for 20 min. The obtained PHEMA single networks were placed in distilled water, which was daily changed until no traces from any residuals were found in the wastewaters, followed by UV-Vis spectroscopy (Jasco V-730 UV/Vis spectrophotometer, Jasco, Tokyo, Japan). HEMA conversion to PHEMA was evaluated to be ~96 ± 2% as determined using wastewater analysis after averaging the results obtained for seven different SNs. The gel fraction, determined gravimetrically using the weights of the PHEMA SNs, dried at room temperature under vacuum, before and after washing, was evaluated to be ~95 ± 3%. 

In the second step, dry PHEMA SNs were immersed in DMAM aqueous solutions with different concentrations (Table 1), containing also 0.1 mol% PEGDA and 0.1 mol% HCHPK. The PHEMA SNs were left to swell for 72 h refrigerated at 4 °C. Then, the swollen SNs were placed under a UV-lamp (365 nm) for 20 min to carry out the in situ DMAM polymerization within the PHEMA SNs. Thus, the formed IPNs were placed in distilled water, which was changed daily, until no traces from any residuals were detected through UV. DMAM conversion to PDMAM, determined through the analysis of the collected wastewaters, was found to be ~92 ± 5% for all IPNs. The exact composition of the PHEMA/PDMAM IPNs, expressed by the PDMAM weight part in the respective IPN, was calculated by using Equation (1):

(1) $$r^{PDMAM}=\frac{m^{PDMAM}}{m^{PHEMA}+m^{PDMAM}}$$

where $m^{PHEMA}$ is the mass of the dry PHEMA SN and $m^{PDMAM}$ is the mass of the PDMAM network determined on the basis of PDMAM conversion. 

For the sake of comparison, PDMAM SNs were synthesized from 1 M DMAM aqueous solution, also containing 0.1 mol% HCHPK and 1 mol% PEGDA. All SNs and IPNs synthesized within this study are summarized in Table 1. The obtained SNs and IPNs were dried and maintained in ambient conditions—25 °C and 45% relative humidity.

### 2.3. Swelling Properties

#### 2.3.1. Equilibrium Swelling Ratio (ESR)

ESR was determined by immersing preliminary weighted dry disk-shaped IPN and SN samples in water and in PBS, respectively. The weight of each sample was measured upon swelling until reaching constant value, and this value was used to calculate ESR using Equation (2).

(2) $$ESR=\frac{m^{swollen}-m^{dry}}{m^{dry}}$$

where $m^{swollen}$ and $m^{dry}$ are the weights of the samples in their dry and equilibrium swollen state, respectively. Results were obtained after averaging the values obtained in three independent measurements for each IPN or SN composition and standard errors are also provided. 

#### 2.3.2. Ionic Strength Responsiveness

Dry disk-shaped IPN and SN samples were immersed for 24 h in NaCl aqueous solutions with different ionic strengths, namely 0.001 M, 0.01 M, 0.1 M, 1 M, 2 M, and 5 M. Their swelling ratios (SR) were calculated using Equation (2), where $m^{swollen}$ is the weight of the respective sample after 24 h of swelling in the respective NaCl aqueous solution.

#### 2.3.3. pH Responsiveness

Dry disk-shaped IPN and SN samples were immersed for 24 h at 0.1 M citrate buffer with different pH values ranging from 4 to 8. Their swelling ratios were calculated using Equation (2), where $m^{swollen}$ is the weight of the respective sample after 24 h of swelling at the respective pH.

#### 2.3.4. Temperature Responsiveness

Dry disk-shaped IPN and SN samples were immersed in water at different temperatures, ranging from 20 to 55 °C, for 6 h. Their swelling ratios were calculated using Equation (2), where $m^{swollen}$ is the weight of the respective sample after 6 h of swelling at the respective temperature. 

#### 2.3.5. Scanning Electron Microscopy (SEM) 

The morphology of fractured surface of dry IPN and SN samples was examined using a scanning electron microscope (JSM-5510, JEOL, Tokyo, Japan) operating at 10 kV. Prior to the observations, the samples surface was coated with gold for 30 s using a sputter-coater (JSC 1200, JEOL, Japan) under argon atmosphere.

#### 2.3.6. Energy-Dispersive X-ray (EDX) Spectroscopy 

The EDX analysis of dry DXP-loaded samples was carried out using scanning electron microscope Lyra 3 XMU (Tescan, Brno, Czech Republic), operating at 10 kV, coupled with an electron backscatter diffraction detector and EDX analysis system (Quantax 200, Bruker, Billerica, MA, USA). Prior to the analysis, the samples were covered with carbon (~10 nm thickness).

#### 2.3.7. X-ray Diffraction (XRD) 

Siemens D500 diffractometer (Munich, Germany) with secondary monochromator and Cu-Kα radiation was used to obtain X-ray diffractograms of the samples in their dry state in the 2θ range 10–80° with a step of 0.05° and count time of 1.0 s.

#### 2.3.8. Temperature-Modulated Differential Scanning Calorimetry (TMDSC)

TMDSC was performed using DSC apparatus Q200, TA instruments, New Castle, DE, USA. Room-conditioned samples were tested using Tzero aluminum pans (TA instruments) in the temperature range from −50 to 250 °C with 5 °C min^−1^ heating rate, modulation amplitude of 1 °C, and a period of 60 s under nitrogen flow (50 mL min^−1^). 

#### 2.3.9. Attenuated Total Reflectance–Fourier Transform Infrared Spectroscopy (ATR-FTIR)

All samples were studied in their dry state using IRAffinity-1 Shimadzu Fourier Transform Infrared spectrophotometer with MIRacle Attenuated Total Reflectance Attachment, Kyoto, Japan. The samples were tested without a preliminary preparation.

#### 2.3.10. DXP Loading

Dry disk-shaped IPN and SN samples were immersed in DXP aqueous solutions with different concentrations, namely 25, 12.5, and 1.25 mg/m, for 72 h at 25 °C. Due to the light sensitivity of DXP, the DXP solution and polymer samples, loaded with DXP, were exposed to light only for brief manipulations; thus, the loading process was carried out in a light-protected environment. Entrapment efficiency (EE) and the drug loading (DL) of DXP were calculated by measuring the UV absorbance of the DXP solution left after the loading process. To this purpose, a calibration curve of DXP in water was obtained by measuring the absorbance at 242.5 nm of three independently prepared series of DXP aqueous solutions. The linear regression of the DXP calibration curve in water (Figure S1) is as follows:

(3) $$Y=0.00164+X\times 0.02478(R^{2}=0.99996)$$

where X and Y are, respectively, the DXP concentration, and its UV absorbance measured at 242.5 nm.

EE and DL of DXP were calculated using Equations (4) and (5), respectively:

(4) $$EE=\frac{m_{sample}^{DXP}}{m_{total}^{DXP}}\times 100\%$$

(5) $$DL=\frac{m_{sample}^{DXP}}{m_{nonloaded}^{sample}}\times 100\%$$

where $m_{sample}^{DXP}$ is the amount of DXP loaded in one polymer piece, $m_{total}^{DXP}$ is the initial amount of DXP in the loading solution, and $m_{nonloaded}^{sample}$ is the weight of the dry polymer piece before DXP loading.

#### 2.3.11. DXP Release

The DXP-loaded hydrogels were transferred into sealed containers containing 25 mL of PBS to ensure sink conditions and equilibrated at 32 °C using an orbital shaker (100 rpm). At defined time intervals, 0.5 mL aliquots were withdrawn from the release media and after appropriate dilution, the UV absorbance was measured at 242.5 nm. The amount of the released DXP was calculated using the calibration curve for DXP obtained in PBS (Figure S2) (Equation (6)):

(6) $$Y=2.02\times 10^{-4}+X\times 0.02509\;(R^{2}=0.99994)$$

All measurements were triplicated. 

#### 2.3.12. DXP Release Kinetics

DXP release profiles were analyzed using the main kinetic models, namely: 

Zero-order (ZO)—this is concentration-independent model where the rate of drug release is only a function of time:

(7) $$Q_{t}=Q_{0}+k_{0}\times t$$

where $Q_{t}$ and $Q_{0}$ are, respectively, the drug amounts released at moment 0 (starting point) and at time t (in hours) from the beginning of the drug release experiment; and $k_{0}$ is a kinetic constant.

First order (FO)—this is the concentration-dependent model where the drug release rate is proportional to the drug concentration:

(8) $$\log Q_{t}=\log Q_{0}-k_{1}\times \frac{t}{2.303}$$

where $k_{1}$ is a kinetic constant.

Higuchi model (HM)—a model developed to describe the drug release from a matrix system, where the amount of the released drug is proportional to the square root of time:

(9) $$Q_{t}=k_{H}\times t^{0.5}$$

where $k_{H}$ is a kinetic constant.

Korsmeyer–Peppas model (KP)—this model is developed to describe the release of a drug molecule from a polymeric matrix, such as a hydrogel.

(10) $$\frac{Q_{t}}{Q_{\infty }}=k_{KP}\times t^{n}$$

where $Q_{\infty }$ is the amount of drug loaded in the sample, $k_{KP}$ is a kinetic constant, and n is a diffusional exponent that allows for determination of the type of drug release mechanism: when n ≤ 0.45, the drug release is realized through Fickian diffusion mechanism; when 0.45 ≤ n ≤ 0.89, the drug release follows the abnormal (non-Fickian) diffusion mechanism; and when n > 0.89, the drug release follows a complex transport mechanism (super-case-II transport)

## 3. Results and Discussion

### 3.1. Swelling Properties

Hydrogels’ swelling is one of the most important parameters that define their application as drug delivery systems. As body fluids have a defined pH, temperature, and ionic strength, we studied the effect of these media characteristics on the IPN swelling behavior as they are also expected to influence their drug delivery performance. 

**Figure 1:**
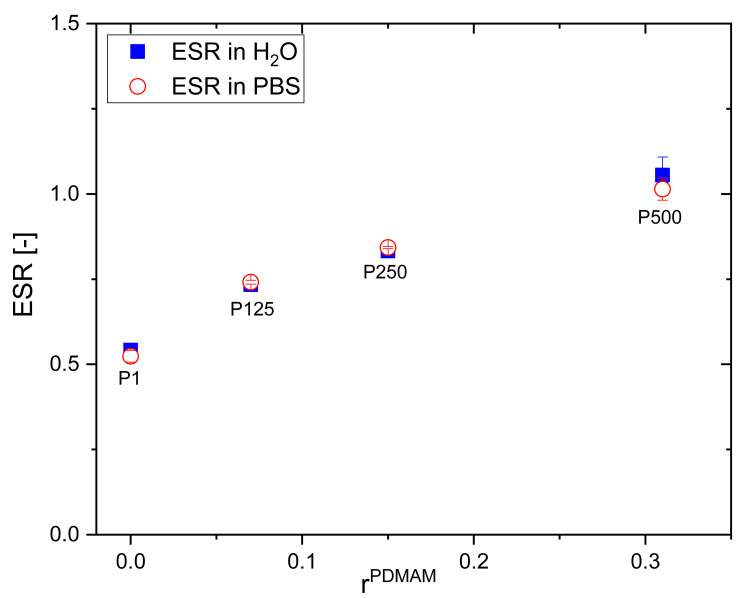
ESR of PHEMA SN and PHEMA/PDMAM IPNs in water and in PBS as function of PDMAM weight part.

The swelling behavior of PHEMA/PDMAM IPNs depends on its composition, defined by the PDMAM weight fraction (Figure 1): the increase in the PDMAM content results in a significantly enhanced ESR of the respective IPN-ESR, increases almost twice from 0.54 ± 0.01 for PHEMA SN (r^PDMAM^ = 0) to 1.06 ± 0.05 for the IPN sample with the highest PDMAM weight part (P500, r^PDMAM^ = 0.31). This can be attributed to the highly pronounced hydrophilicity of the PDMAM, well demonstrated by the high swelling capacity of PDMAM SN, synthesized with 4 mol% PEGDA as a crosslinking agent, which was evaluated to be 9.65 ± 0.22. The incorporation of such a highly hydrophilic component into the IPNs results in an increase in the overall swelling capacity of the resulting IPNs. 

A similar study showed that the swelling capacity of PHEMA can be improved through its copolymerization with a hydrophilic co-monomer such as vinylpyrrolidone (VP) [16]. Xu et al. reported an increase in the ESR of the neat PHEMA from ~45% to ~65% observed for the poly(HEMA-co-VP) hydrogels with 25% PVP content. Similarly, the PVP component increases the ESR of PHEMA/PVP IPNs from 49.1 to 72.3% upon increasing the PVP content from 25.3 to 78.7% [17]. While the PVP inclusion enhances the swelling capacity of PHEMA hydrogels with ~20%, the PDMAM component inclusion could increase almost twice the PHEMA/PDMAM IPN swelling ability, which is also expected to enhance their drug loading capacity.

**Figure 2:**
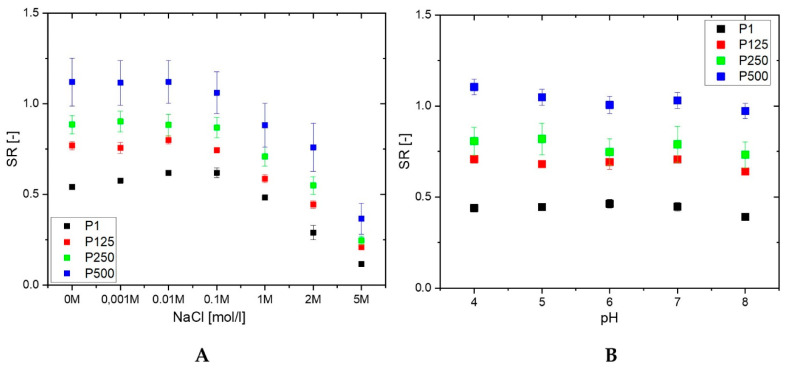
SR of PHEMA SN and PHEMA/PDMAM IPNs as a function of the ionic strength (**A**) and pH (**B**) of the media.

The swelling ratio of PHEMA/PDMAM IPNs in water and PBS with an ionic strength of 0.1 M is the same within the experimental error (Figure 1). However, upon further increasing the ionic strength of the solution, using NaCl aqueous solutions with different concentrations, the SR decreases for both PHEMA SN and the PHEMA/PDMAM IPNs (Figure 2A). Thus, ionic strengths below 0.1 M do not affect the SR, but above this value, the swelling ratio decreases. Since neither PHEMA nor PDMAM bear ionizable side groups, this SR decrease cannot be explained as a polyelectrolyte effect [18]. However, this observation is in good agreement with other works related to PHEMA-based materials [19] whereupon increasing the salt concentration, a decrease in the osmotic pressure is observed, which leads to solvent efflux from the hydrogel to the media and the SR decreases. 

The drug release rate is expected to be influenced by the swelling capacity of the hydrogel, especially having in mind that sweat has an ionic strength equal to 0.1 M NaCl. Thus, the observed dependence of the PHEMA/PDMAM IPN swelling ratio on the medium ionic strength means that the drug release rate will not be strongly affected by the person-to-person variations in sweat composition. Sweat’s Na^+^ and Cl^−^ concentrations, although varying with thermal stress, body temperature, and sweat production rate [20], remain around 42.9 ± 18.7 mmol·L^−1^ and 32.2 ± 15.6 mmol·L^−1^, respectively [21]. 

None of the PHEMA/PDMAM IPNs show a pH dependence of their SR in the pH range between 4 and 8 (Figure 2B) at an ionic strength of 0.1 M. However, the different hydrophilicity defined by the PDMAM weight ratio in the IPNs is well seen in Figure 2B: the higher the PDMAM content, the higher the respective swelling ratio. 

**Figure 3:**
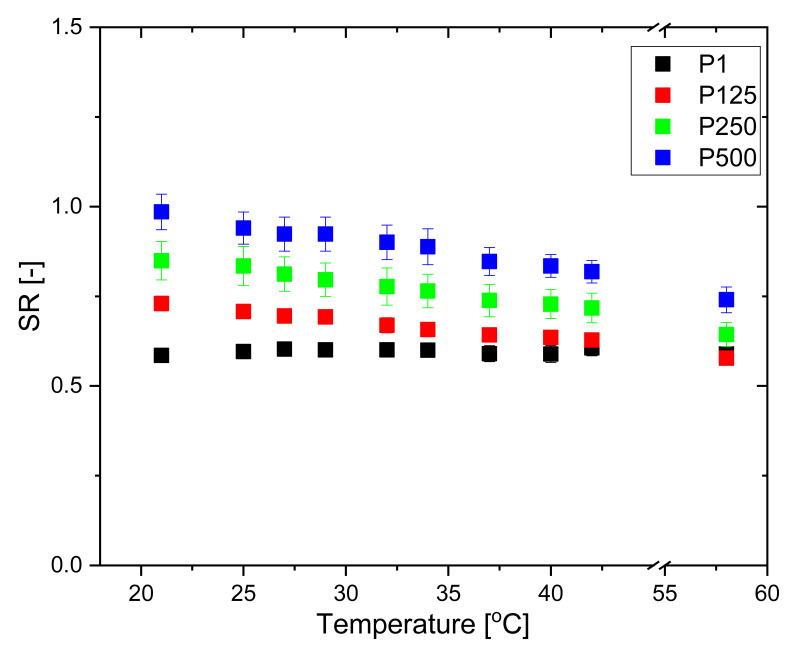
SR of PHEMA SN and PHEMA/PDMAM IPNs in water as a function of temperature.

The PHEMA SN does not exhibit any dependence on temperature, and its SR remains constant as temperature increases. In contrast, the PHEMA/PDMAM IPNs exhibit a slight temperature responsiveness: the SR decreases linearly with the temperature increase and this effect is more pronounced for higher PDMAM weight ratios (Figure 3). 

It is well known that the temperature responsiveness of polymers is mainly governed by the hydrophobic/hydrophilic balance of the pendant groups. The present system is composed of PHEMA, bringing pendant –CH_3_ and –CH_2_CH_2_OH groups, and PDMAM with pendant –N(CH_3_)_2_ groups. These hydrophobic side groups give rise to hydrophobic interactions, which are known to increase with increasing temperature. Thus, the PHEMA/PDMAM IPNs’ SR decrease upon a temperature increase is related to the formation of hydrophobic clusters between polymeric side groups, which present additional physical junctions, reducing their swelling ability. Similarly, in other systems, the distribution of the pendant groups with the ability to form hydrophobic domains within the IPNs is random rather than alternating [22]. The observed temperature dependence of SR is probably defined by the IPNs’ entangled structure, which additionally limits the SR and hence the temperature responsiveness. The more crosslinked the polymers are, the less sharp is their temperature-induced transition, so the IPNs’ SR decrease in Figure 3 is linear.

### 3.2. DXP Loading 

The entrapment efficiency (EE) and drug loading (DL) of DXP into PHEMA/PDMAM IPNs were determined using DXP solutions with three different concentrations, namely 25, 12.5, and 1.25 mg/mL, while keeping the drug/polymer ratio constant at 1:7. 

**Figure 4:**
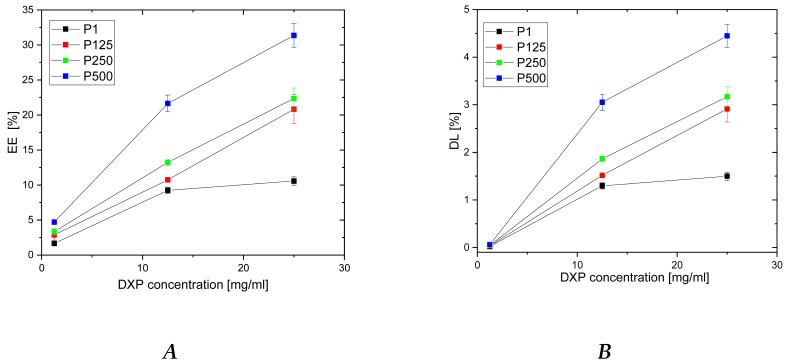
Dependence of DXP EE (**A**) and DL (**B**) in PHEMA SN and PHEMA/PDMAM IPNs as a function of the DXP concentration in the loading solution.

For PHEMA SNs, the EE and DL level off at a DXP concentration of 12.5 mg/mL, with the maximum reached values for both parameters being, respectively, 7.5% and 1.2% (Figure 4). The EE and DL plateau obtained for PHEMA SN could be explained by its comparatively low SR in water, which limits more DXP from entering the PHEMA SN.

For all IPNs, the higher the DXP concentration, the higher the EE and the DL are (Figure 4). Moreover, a clear dependence of EE and DL is observed on PDMAM content, which is more clearly presented in Figure S3. Thus, the sample with the highest PDMAM content (P500, r^PDMAM^ = 0.31) has EE~32%, which is nearly four times greater than the EE for PHEMA SN, and the highest DL, among all samples in this study, is ~4.5%. This PDMAM content dependence of EE and DL could be related to the data obtained for ERS, which also increases with PDMAM content (Figure 1). The direct correlation between EE and ESR, as well as between DL and ESR, is presented in Figure S4. Both parameters, EE and DL, increase almost linearly as ESR increases, which, on the other hand, increases with the PDMAM weight ratio increase. All these data suggest that DXP loading into SNs and IPNs takes place through diffusion rather than through DXP active transport.

### 3.3. Scanning Electron Microscopy (SEM)

**Figure 5:**
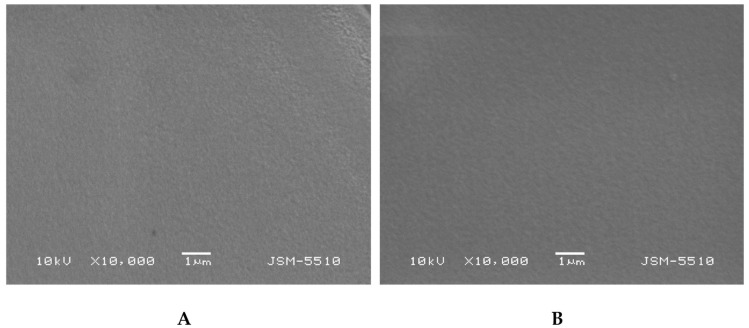

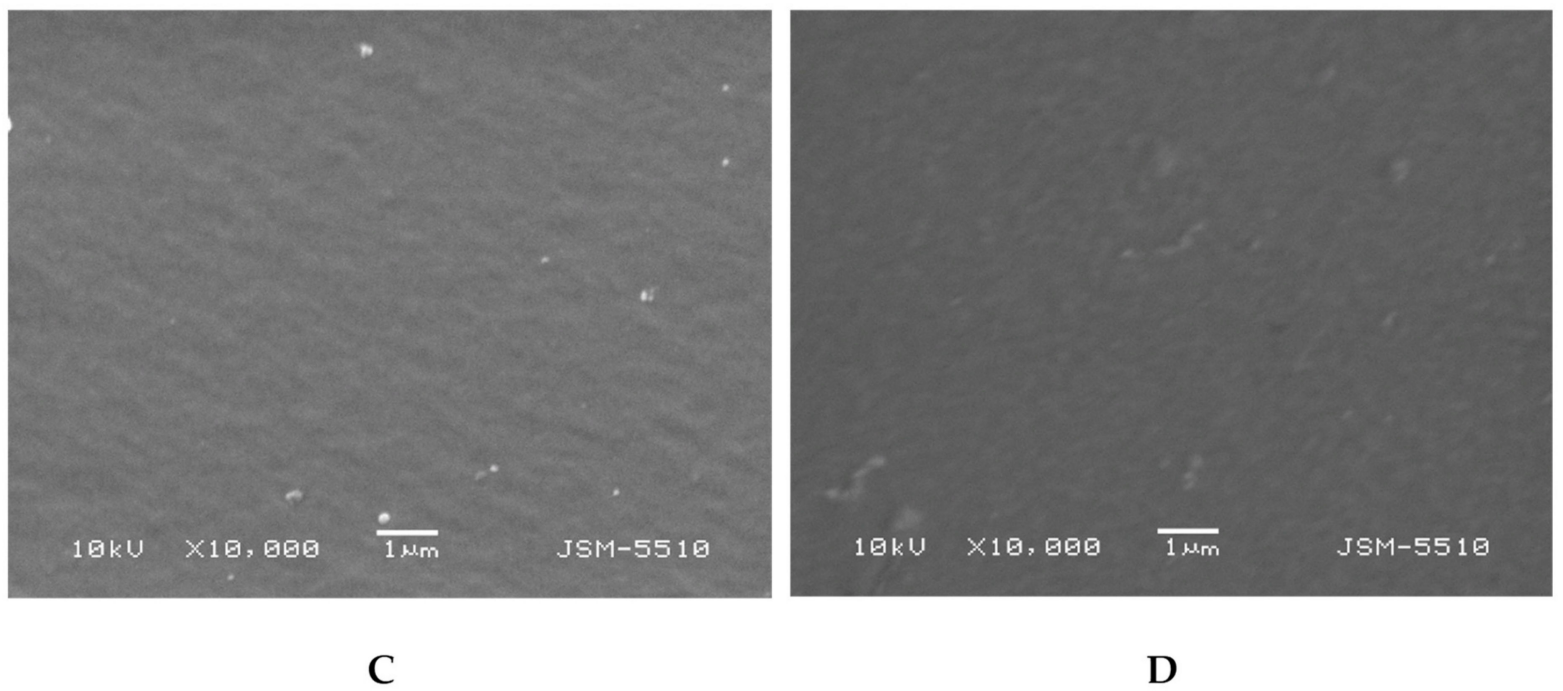
Morphology of fractured surfaces of samples P1 (**A**) and P1 when loaded with DXP (**B**) as well as of P500 (**C**) and P500 loaded with DXP (**D**).

The morphology of the PHEMA/PDMAM IPNs as well as of PHEMA SNs was studied using SEM before and after loading with DXP (Figure 5). While PHEMA SNs have quite a smooth fractured surface that does not change upon DXP loading (Figure 5A,B), the morphology of the IPN with the highest PDMAM content (P500, r^PDMAM^ = 0.31) appears to be coarser most probably due to the inclusion of the second network from PDMAM. 

Typically, IPNs tend to phase-separate, as shown in our previous studies [23], but this trend is not clearly seen here, most probably due to the better miscibility between both constituent networks. The DXP loading does not significantly change the IPN morphology and no clear indication for DXP crystallite formation is seen, which supports the DXP solubilization within the polymer matrices.

### 3.4. EDX Analysis

**Figure 6:**
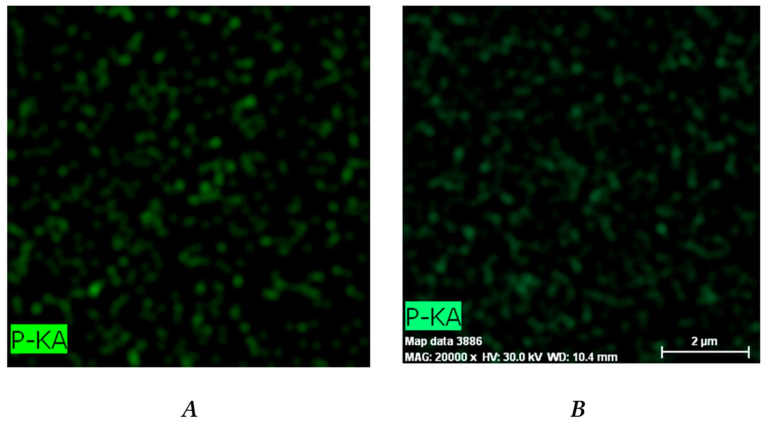

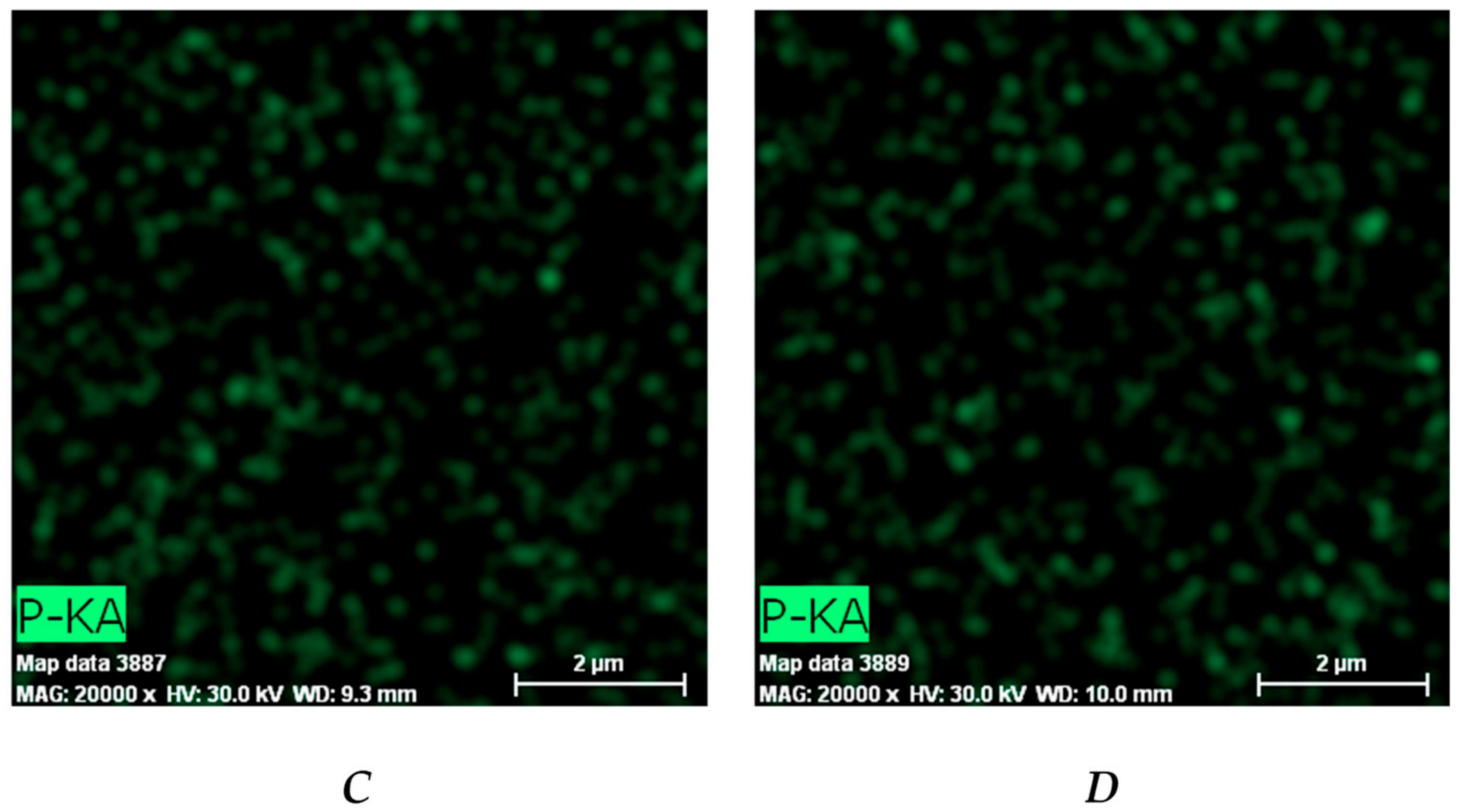
Distribution of the phosphorous within the DXP-loaded samples P1 (**A**), P125 (**B**), P250 (**C**), and P500 (**D**) as revealed by EDX.

EDX analysis was used to evaluate the DXP distribution within the PHEMA/PDMAM IPNs. Phosphorous from the DXP molecules (C_22_H_28_FNa_2_O_8_P) was used as a marker to illustrate the drug dispersion as it is not present in any of the IPN constituents. Phosphorus mapping (Figure 6) confirms the successful DXP loading in the PHEMA/PDMAM IPNs and reveals its distribution within the whole samples. 

The relative percentage of phosphorus in the three IPN samples is significantly higher as compared to the phosphorus determined in PHEMA SNs (Figure S5), indicating that a higher amount of DXP is loaded into the IPNs as compared to the PHEMA SNs. This result is in agreement with the results about EE and DL presented in Figure 4. 

### 3.5. X-ray Diffraction (XRD) 

**Figure 7:**
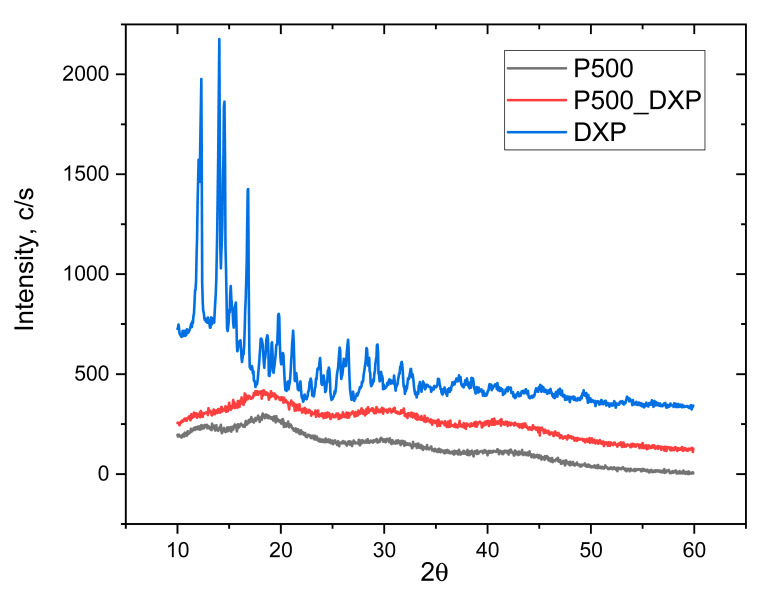
XRD patterns of pure DXP, and non-loaded and DXP-loaded IPN P500 (r^PDMAM^ = 0.31), all in dry state.

XRD analysis was used to evaluate if, when loaded, the drug remains crystalline or is amorphized after the loading procedure. The XRD patterns of the pure DXP and DXP-loaded IPN sample P500 (drug loaded in 25 mg/mL of DXP solution) are shown in Figure 7. The sample P500 was chosen since it is the sample with the highest DXP DL (~4.5%). Pure DXP is a highly crystalline substance with two distinctive peaks at 12° and 25°. When DXP is loaded in P500 IPN (r^PDMAM^ = 0.31), no crystal peaks are detected in the respective diffractogram and only an amorphous halo is observed. This means that DXP is completely solubilized at the molecular level within the P500 IPN structure. This result is in good agreement with other works [24] and it is in line with the observations from SEM and EDX obtained within this study. 

### 3.6. ATR-FTIR

ATR-FTIR spectra of PHEMA/PDMAM IPNs, non-loaded and DXP-loaded, were obtained to study the drug–polymers interactions. At the spectrum of sample P500 and DXP-loaded P500, the bands at 1717, 1456, 1153 and 1022 cm^−1^ can be assigned to the -C=O stretching, -C−O stretching, and -C−H bending of PHEMA, respectively. In the spectrum of DXP, the strong absorption band at 1667 cm^−1^ can be assigned to the -C=O stretching vibration. The bands at 1047 and 1025 cm^−1^ originate from the stretching frequency bands of the phosphate anion (P–O) of dexamethasone sodium phosphate [25].

**Figure 8:**
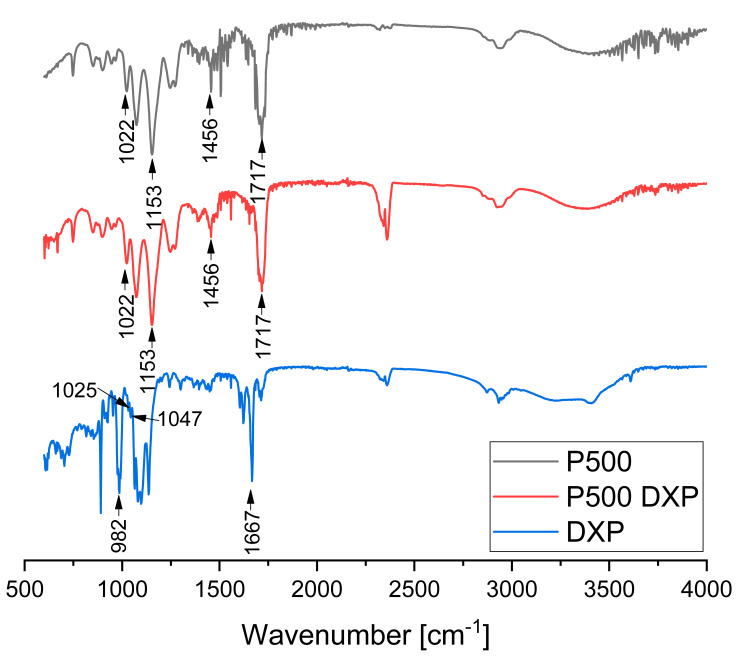
ATR-FTIR spectra of sample P500, DXP-loaded P500, and pure DXP.

The ATR-FTIR spectrum of sample P500, which is the PHEMA/PDMAM IPN with the highest DXP loading, shows complete disappearance of the bands’ characteristic for the pure DXP (Figure 8). At the same time, the position and intensity of the bands, originating solely from P500 (Figure 8 and Figure S6), do not change, which is an indication for the lack of detectable interaction between the DXP and the polymer matrix. Thus, by using IR, it was not possible to detect a clear interaction between the drug and the polymer, and the lack of the bands for the pure DXP points out its amorphization, which is in a good agreement with the XRD data that also showed complete amorphization of DXP. 

### 3.7. Thermal Properties of PHEMA/PDMAM IPNs, Non-Loaded and DXP-Loaded, as Revealed by TMDSC

TMDSC was used to deconvolute the overlapping glass transition (Tg) and water evaporation/enthalpy relaxation in the PHEMA and PDMAM SNs as well as their IPNs (Figure S7), and the obtained Tg values are summarized in Table S2. The obtained PHEMA SN Tg ~69 °C is a little lower as compared to the Tg ~115 °C reported in the literature for neat PHEMA [26]. However, this could be explained by the presence of the crosslinking agent PEGDA, as González-Henríque et al. have reported Tg ~55 °C for PHEMA, crosslinked with 1% PEGDA (Mn = 575) [27]. The Tg of PDMAM SNs was found to be ~43 °C, which is again lower than the reported Tg ~89 °C for neat PDMAM [28]. The same explanation is proposed here, as PDMAM is also crosslinked by PEGDA. For the sake of comparison, the Tg values of neat PEGDA networks were reported to be in the range of −40 to −30 °C [29]. 

**Figure 9:**
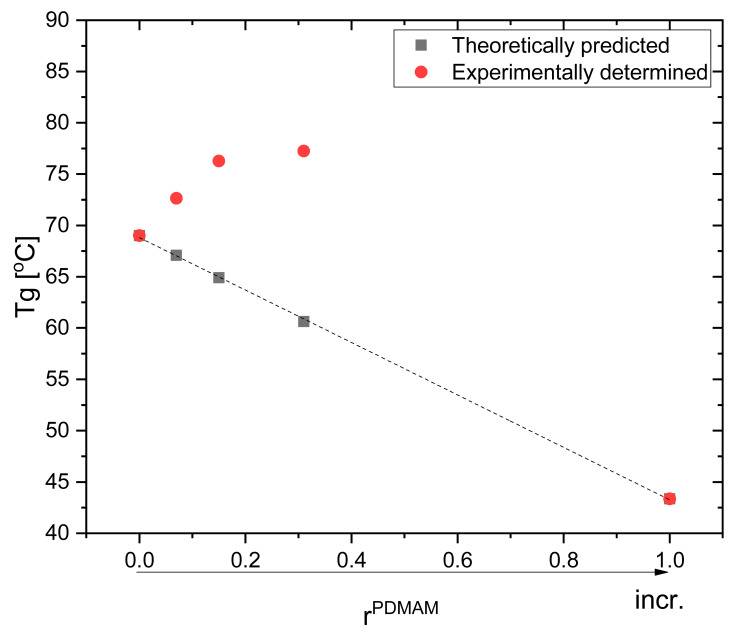
Glass transition temperatures of neat IPNs: as predicted by the Fox equation and the experimentally determined IPNs’ Tgs.

Tgs of PHEMA/PEGDA IPNs are higher as compared to the Tgs of the respective SNs and they increase as r^PDMAM^ increases (Figure 9). Based on the experimentally determined Tgs for PHEMA and PDMAM SNs, the theoretical Tgs of their respective IPNs were calculated in accordance with the Fox equation (additivity law):

(11) $$\frac{1}{T_{g}}=\frac{w_{1}}{T_{g_{1}}}+\frac{w_{2}}{{T_{g}}_{2}}$$

where $T_{g}$ is the glass transition temperature of the respective IPN; and $w_{1}$, $w_{2}$, $T_{g_{1}}$, and $T_{g_{1}}$_,_ are the weight parts and glass transition temperatures of PHEMA and PDMAM SNs, respectively. As it can be seen, the experimentally determined Tgs are higher compared to the theoretically predicted ones, i.e., a positive deviation from the additivity law. Such a positive deviation can be attributed to the additional interlacing and intertwining between both constituent networks in the IPN, which increases the total network density and hence increases their Tgs.

Pure DXP shows two endothermic peaks at ~89 °C and ~205 °C. The first one is most probably due to dehydration of strongly bound water molecules [30], while the second is due to the DXP melting. The latter totally disappears in the thermograms of DXP-loaded SN and IPNs, proving that DXP is fully amorphous when loaded in the polymer matrices. This conclusion is in line with the results from the XRD analysis as well as with the SEM observations, which also indicated the fully amorphous drug in the drug-loaded polymer samples.

**Figure 10:**
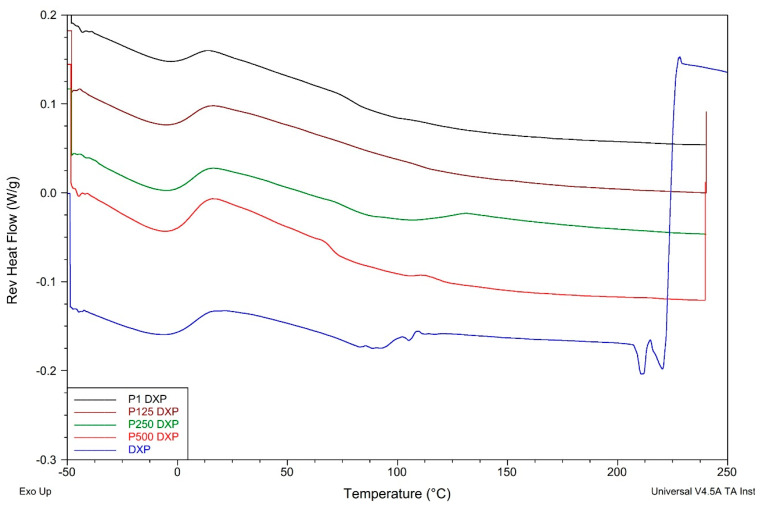
Reversing heat flow thermograms of DXP-loaded PHEMA/PDMAM IPNs and PHEMA SN as well as of pure DXP.

The DXP loading results in an increase in Tgs of all loaded polymer matrices as compared to the respective non-loaded samples (Figure 10, Table S2). Similar behavior could be due to the polymer–drug interaction, which immobilizes the polymer chain segments and thus increases the Tg values. The EDX study revealed a grain-like structure of the DXP loaded into the PHEMA/PDMAM IPNs and these small grains are amorphous according to the DSC and XRD results; however, they represent solid inclusions into the polymer matrix. This structure resembles the structure of polymer nanocomposites where it is known that the polymer chains absorb onto the solid nanoparticles, and this affects their mobility and hence the nanocomposites’ Tg. On the other hand, similar effects are reported for the amorphous indomethacin influence on the PVA copolymers, acting as a delivery system for it, and it is denoted as antiplasticization. The drug’s small amorphous inclusion reduces the free volume and immobilizes the PVA copolymers’ chains, decreasing the true density as well as provoking specific intermolecular interactions [31]. Thus, both mechanisms result in the same Tg dependence on the drug inclusion in the polymer matrices.

### 3.8. DXP Release

**Figure 11:**
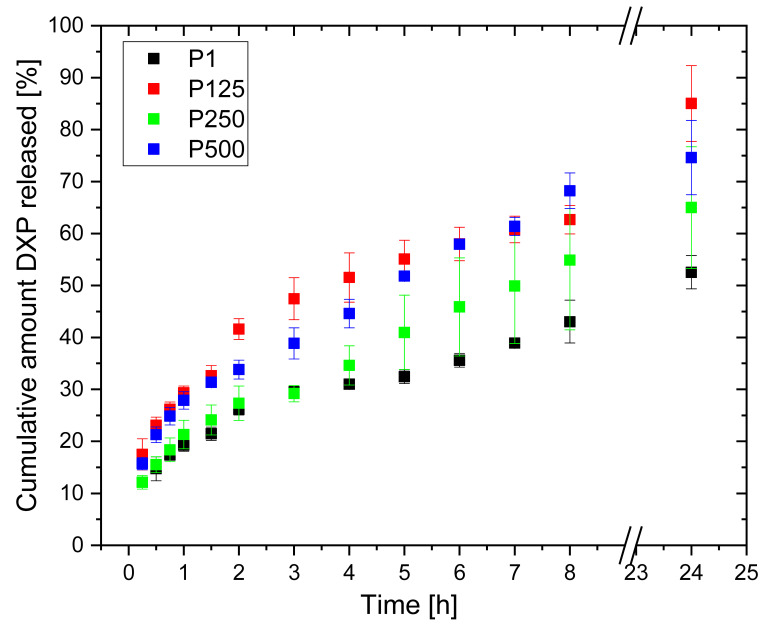
DXP release profiles from PHEMA SN and PHEMA/PDMAM IPNs loaded in 25 mg/mL of DXP aqueous solution.

The DXP release profiles from PHEMA/PDMAM IPNs loaded in 25 mg/mL of DXP aqueous solution (i.e., with the highest load) are presented in Figure 11. All samples gradually release DXP in the first 8 h, and for all of them, up to ~80% DXP is released within the first 24 h. 

No burst effect is observed, meaning that the drug is mostly deposited/loaded within the polymer matrices and not on their surfaces, thus retarding the drug release and avoiding its immediate release. The PDMAM component of the IPNs allows the release of a higher amount of DXP as compared to the neat PHEMA SN. This observation correlates well with the way the IPN composition influences the IPNs’ swelling, which also enhances the drug diffusion. 

The kinetic model’s analysis shows that the Higuchi model best describes the observed DXP release profiles, which means that DXP is released from the IPN hydrogels following Fickian diffusion. The Korsmeyer–Peppas model shows that the diffusional exponents of DXP release depend on the IPN composition as they increase with the PDMAM content, e.g., from n = 0.338 for sample P1 (r^PDMAM^ = 0) to n = 0.421 for sample P500 (r^PDMAM^ = 0.31) (Table 2). 

**Figure 12:**
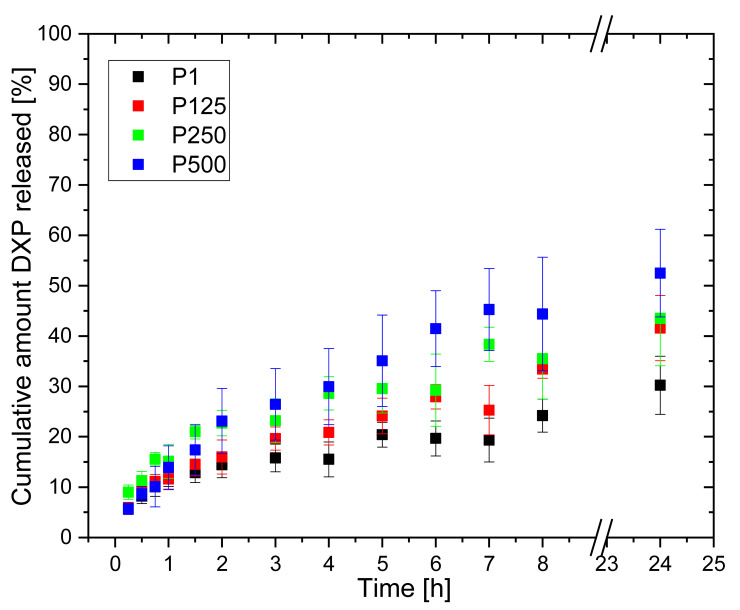
DXP release profiles from PHEMA SN and PHEMA/PDMAM IPNs loaded in 12.5 mg/mL of DXP water solution.

The DXP release from the same polymer matrices, but loaded in 12.5 mg/mL of aqueous DXP solution (Figure 12), show similar profiles, although DXP release is slower—the maximal amount of DXP released is ~50% for 24 h. Again, the Higuchi model best describes the DXP release (Table 3); however, the diffusional exponents obtained using the Korsmeyer–Peppas model are higher and vary from n = 0.357 for sample P1 (r^PDMAM^ = 0) to n = 0.550 for sample P500 (r^PDMAM^ = 0.31). Generally, values for n ≤ 0.45 are indicative for hampered Fickian diffusion [32] where physical barriers such as hydrophobic clusters increase the tortuosity of the drug molecules’ motion toward media, thus retarding its release. Such hydrophobic clusters can be formed by -CH_3_ and -C_2_H_4_- groups of PHEMA and -CH_3_ groups of PDMAM. It can be expected that the increase in PDMAM content will additionally decrease n through the formation of more hydrophobic clusters, but as the PDMAM content is increased, this increases the hydrophilicity and swelling ratio of the respective IPNs. The increased water uptake facilitates the dissolution and release of the water-soluble DXP; thus, a Fickian diffusion (n~0.45) is observed. In addition, the diffusional exponents values increase as the ESR of the respective IPN increases (Figure S8).

As the results show, sample P500 (r^PDMAM^ = 0.31) exhibits the most satisfying drug delivery properties—the highest loading capacity with DXP and the longest release profile with approx. 70% of DXP release in 24 h without. The analysis with the kinetic models shows that DXP is released through Fickian diffusion (n = 0.421) and this is nearly linear for the first eight hours, i.e., zero-order release kinetics is observed. 

## 4. Conclusions

This study reveals the potential of PHEMA/PDMAM IPNs as dermal delivery systems of DXP. The swelling properties of the IPNs depend on the ratio between the constituents, i.e., the IPN composition is a key factor that defines their properties. The higher the PDMAM content is, the higher their equilibrium swelling ratios both in water and in PBS, reaching ~1.1, compared to ~0.5 for PHEMA SNs. PHEMA/PDMAM IPNs exhibit temperature and ionic strength responsiveness, and expectedly, they are not pH-responsive. The DXP loading in PHEMA/PDMAM IPNs is diffusion-controlled and the PDMAM content as well as DXP concentration in the loading solution are the key factors increasing the DXP entrapment efficiency up to ~30%. DXP becomes amorphous upon loading into the PHEMA SNs, as well as in PHEMA/PDMAM IPNs. The DXP is released with no “burst” effect and reaches ~70% for 24 h depending on the PDMAM content. The diffusional exponent obtained from the Korsmeyer–Peppas model is between n = 0.357 and 0.550, suggesting that DXP is released through Fickian diffusion. This study reveals the advantages that the IPN approach provides when developing novel drug delivery systems, as this approach allows for the proper choice of polymeric components as well as the ratio between them, making them key parameters to determine the drug release profiles. Future research will reveal if PHEMA/PDMAM IPNs have suitable mechanical properties for dermal application, i.e., softness and elasticity. Moreover, the same system will be evaluated as a carrier for hydrophobic drugs like dexamethasone and capsaicin, and the in vitro cytotoxicity will be studied. 

## Figures and Tables

**Table 1:** PHEMA/PDMAM IPNs and SNs of PHEMA and PDMAM.

Sample Designation	C^DMAM^ * (mol/L)	$r^{PDMAM}$ **
P1	PHEMA SNs	0
P125	1.25	0.07
P250	2.50	0.15
P500	5.00	0.31
PDMAM	PDMAM SNs	1

* Concentration of DMAM monomer during the 2nd step of the IPNs’ preparation. ** PDMAM weight part in the respective IPN, calculated using Equation (1).

**Table 2:** Kinetics models applied to the DXP release profiles obtained within this study for PHEMA SNs and PHEMA/PDMAM IPNs loaded in 25 mg/mL of DXP aqueous solution.

	P1			P125			P250			P500		
Zero order	k_0_	R^2^		k_0_	R^2^		k_0_	R^2^		k_0_	R^2^	
	0.106	0.930		0.241	0.833		0.228	0.973		0.388	0.989	
First order	k_1_	R^2^		k_1_	R^2^		k_1_	R^2^		k_1_	R^2^	
	−0.046	0.939		−0.09	0.959		−0.074	0.978		−0.097	0.985	
Higuchi	k_H_	R^2^		k_H_	R^2^		k_H_	R^2^		k_H_	R^2^	
	0.32	0.981		0.741	0.973		0.684	0.994		1.28	0.992	
Korsmeyer-Peppas	k_KP_	n	R^2^	k_KP_	n	R^2^	k_KP_	n	R^2^	k_KP_	n	R^2^
	0.193	0.338	0.987	0.266	0.358	0.991	0.196	0.386	0.979	0.263	0.421	0.986

**Table 3:** Kinetics models applied to the DXP release profiles obtained within this study for PHEMA SN and PHEMA/PDMAM IPNs loaded in 12.5 mg/mL of DXP aqueous solution.

	P1			P125			P250			P500		
Zero order	k_0_	R^2^		k_0_	R^2^		k_0_	R^2^		k_0_	R^2^	
	0.046	0.871		0.073	0.937		0.108	0.873		0.229	0.975	
First order	k_1_	R^2^		k_1_	R^2^		k_1_	R^2^		k_1_	R^2^	
	0.08	0.887		−0.142	0.943		−0.211	0.928		−0.494	0.969	
Higuchi	k_H_	R^2^		k_H_	R^2^		k_H_	R^2^		k_H_	R^2^	
	0.141	0.956		0.224	0.981		0.319	0.969		0.663	0.982	
Korsmeyer-Peppas	k_KP_	n	R^2^	k_KP_	n	R^2^	k_KP_	n	R^2^	k_KP_	n	R^2^
	0.142	0.357	0.988	0.151	0.426	0.991	0.202	0.408	0.998	0.179	0.550	0.989

## Data Availability

The raw/processed data required to reproduce these findings cannot be shared at this time, as the data also form part of an ongoing study.

## Supplementary Materials

The following supporting information can be downloaded at https://www.mdpi.com/article/10.3390/pharmaceutics15092328/s1, [33,34], Table S1. Chemical formulas and role of the reagents used within this study; Table S2. Tg of non-loaded and DXP-loaded PHEMA and PDMAM SNs as well as of their IPNs; Figure S1. Calibration curve of DXP in water; Figure S2. Calibration curve of DXP in PBS; Figure S3. Dependence of DXP EE and DL in PHEMA SN and PHEMA/PDMAM IPNs as a function of the PDMAM content into the IPNs, defined by r^PDMAM^; Figure S4. Correlation between (A) EE and ESR of the respective PHEMA/PDMAM IPNs and (B) DL from and ESR of the respective PHEMA/PDMAM IPNs. The PHEMA SN has the lowest ESR and it is also presented; Figure S5. EDX spectra of DXP-loaded samples P1 (A), P125 (B), P250 (C), and P500 (D); Figure S6. ATR-FTIR spectra of PHEMA SN (P1), PDMAM SN, and their IPN with composition IPN P500 (r^PDMAM^ = 0.31); Figure S7. Reversing heat flow TMDSC thermograms of dry PHEMA and PDMAM SNs as well as for their IPNs; Figure S8. Correlation between diffusional exponents (n) of DXP release from PHEMA/PDMAM IPNs and their respective ESR.

## Abbreviations

DL	drug loading;
DMAM	N,N-Dimethylacrylamide;
DSC	Differential scanning calorimetry;
DXP	Dexamethasone sodium phosphate;
EDX	Energy-dispersive X-ray (EDX) spectroscopy;
EE	entrapment efficiency;
ESR	Equilibrium swelling ratio;
HCHPK	1-Hydroxycyclohexyl phenyl ketone;
HEMA	2-Hydroxyethyl methacrylate;
IPNs	Interpenetrating polymer networks;
PDMAM	poly(N,N′-dimethylacrylamide);
PEGDA	poly(ethylene glycol) diacrylate;
PHEMA	poly(2-hydroxyethyl methacrylate);
poly(HEMA-co-VP)	poly[(2-hydroxyethyl methacrylate)-co- vinylpyrrolidone];
PVA	poly(vinyl alcohol);
PVP	polyvinyl pyrrolidone;
SEM	Scanning electron microscopy;
SNs	Single networks;
SR	Swelling ratios;
Tg	glass transition;
TMDSC	Temperature-modulated differential scanning calorimetry;
VP	vinylpyrrolidone;
XRD	X-ray diffraction;
