# Supplementary material for: Interpenetrating Polymer Networks of Poly(2-hydroxyethyl methacrylate) and Poly(N, N-dimethylacrylamide) as Potential Systems for Dermal Delivery of Dexamethasone Phosphate
Source: Pharmaceutics. 2023 Sep 15;15(9):2328. doi: 10.3390/pharmaceutics15092328 (PMC10538039; doi:10.3390/pharmaceutics15092328)
Supplement: Supplementary file 1 [file pharmaceutics-15-02328-s001.zip › pharmaceutics-2576578-supplementary.pdf]

Supplementary information

Table S1. Chemical formulas and role of the reagents used within the study

Name of the reagent	Chemical formula	Role
2-Hydroxyethyl methacrylate (HEMA)	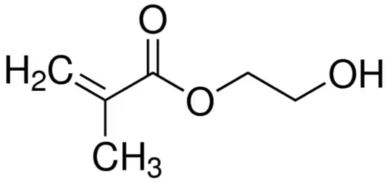	Monomer 1 (used for preparation of the first single network)
N,N-Dimethylacrylamide (DMAM)	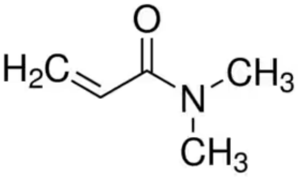	Monomer 2 (used for preparation of the second single network in the IPNs)
Poly(ethylene glycol) diacrylate (PEGDA, Mn=575)	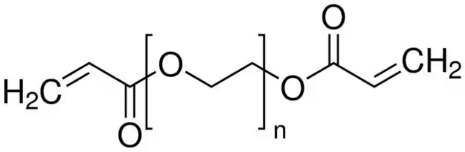	Crosslinking agent for both PHEMA and PDMAM networks
1-Hydroxycyclohexyl phenyl ketone(HCHPK)	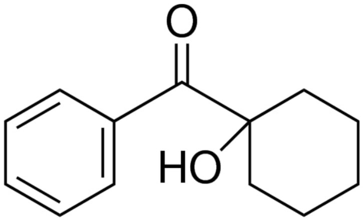	UV-photoinitator
Dexamethasone Sodium Phosphate (DXP)	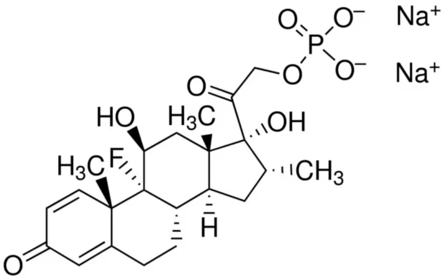	Drug for treatment of arthritis

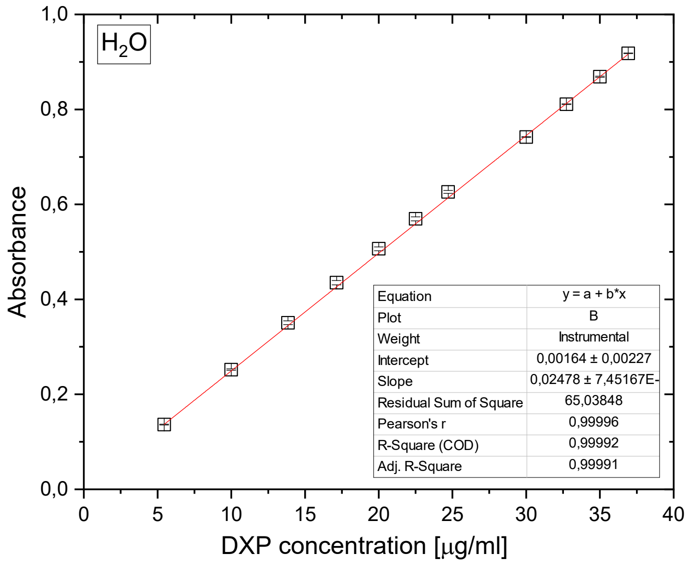

Figure S1. Calibration curve of DXP in water

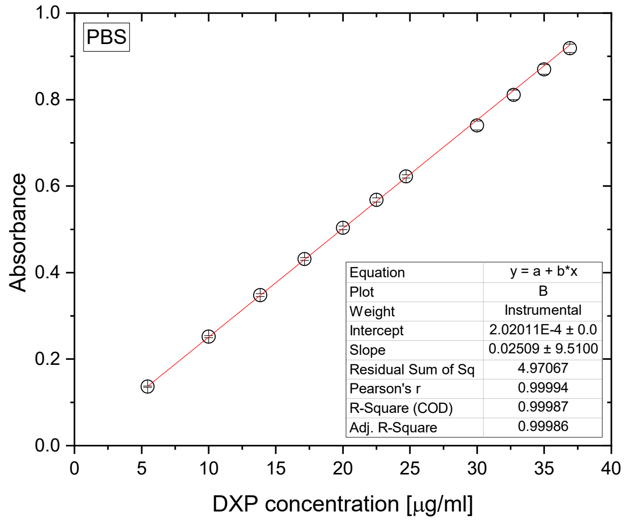

Figure S2. Calibration curve of DXP in PBS

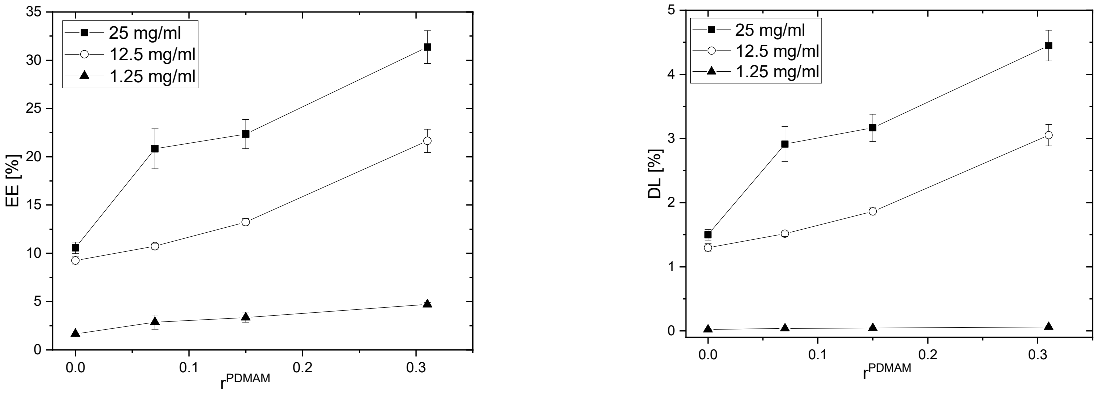

Figure S3. Dependence of DXP EE and DL in PHEMA SN and PHEMA/PDMAM IPNs as a function of the PDMAM content into the IPNs, defined by r^{PDMAM}.

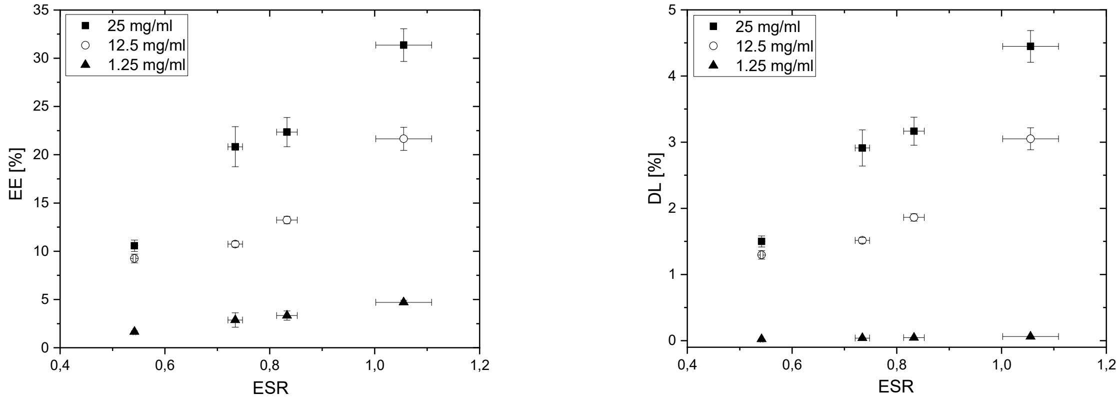

A *B*

Figure S4. Correlation between (**A**) EE and ESR of the respective PHEMA/PDMAM IPNs and (**B**) DL from and ESR of the respective PHEMA/PDMAM IPNs. The PHEMA SN has the lowest ESR and it is also presented.

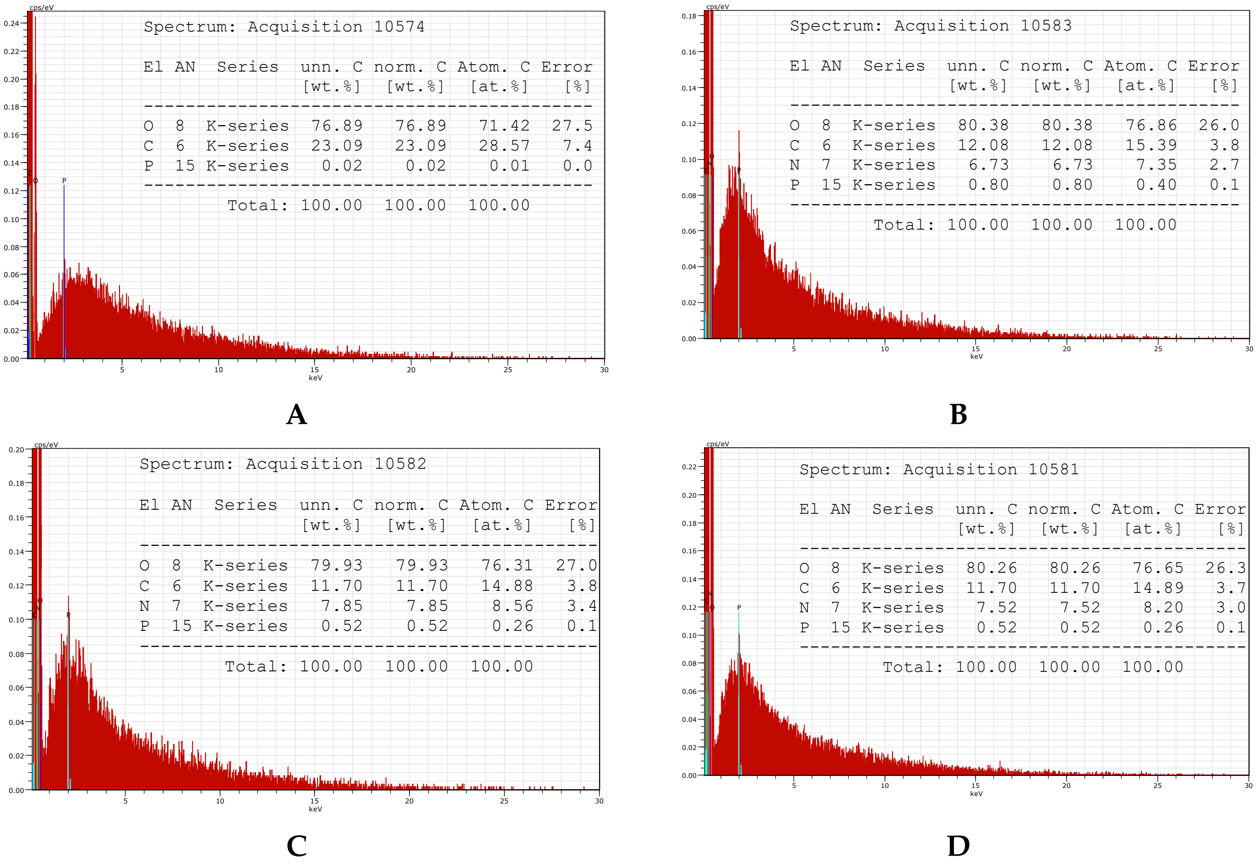

Figure S5. EDX spectra of DXP loaded samples P1 (**A**), P125 (**B**), P250 (**C**) and P500 (**D**)

ATR-FTIR of PHEMA SN, PDMAM SNs as well as of their IPN

ATR-FTIR spectra of non-loaded and DXP-loaded PHEMA/PDMAM IPNs as well as of PHEMA and PDMAM SNs were obtained in order to detail study the interaction between both constituents of the IPNs as well as the drug-polymers interactions.

In the spectrum of PHEMA SN (P1, Figure S6) the characteristic band at 1717 cm^{-1} are assigned to -C=O stretching. In addition, the bands at 1153, 1456, and 2943 cm^{-1} are assigned to -C−O stretching, -C−H bending, and $-CH_2-$ group, respectively. The band at 1022 cm^{-1} in the same spectrum originates from the -C-O bending.[1]

In the spectrum of PDMAM SN (P1, Figure S6), the band at 1653 cm^{-1} is assigned to the -C=O group stretching vibration. The band at 1616 cm^{-1} is attributed to the C=O stretching vibrations (amide I). The band at 1498 cm^{-1} originates from the combination of the -N-H bending and the -C-N stretching vibrations of the amide II group, while the bands at 2926 cm^{-1} and 1251 cm^{-1} correspond to the -C-H stretching and -C-H twisting vibrations, respectively.[2]

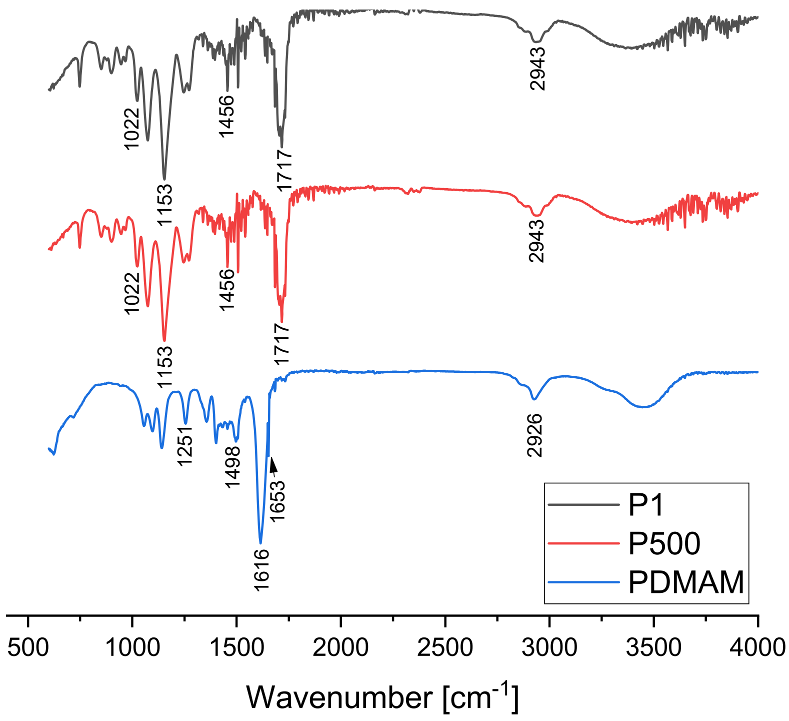

Figure S6. ATR-FTIR spectra of PHEMA SN (P1), PDMAM SN and their IPN with composition IPN P500 (r^{PDMAM}=0.31).

---

[1] Buxadera-Palomero, J.; Fricke, K.; Reuter, S.; Gil, F.J.; Rodriguez, D.; Canal, C. One-Step Liquid Phase Polymerization of HEMA by Atmospheric-Pressure Plasma Discharges for Ti Dental Implants. *Appl. Sci.* **2021**, *11*, 662. https://doi.org/10.3390/app11020662

[2] Lee, J.H., Han, W.J., Jang, H.S. *et al.* Highly Tough, Biocompatible, and Magneto-Responsive Fe_3O_4/Laponite/PDMAAm Nanocomposite Hydrogels. *Sci Rep* **9**, 15024 (2019). https://doi.org/10.1038/s41598-019-51555-5

The bands of PHEMA and PDMAM strongly overlap and in the spectrum of the IPN sample P500 (r^{PDMAM}=0.31) and as there is no change in the position or intensity of the bands in this spectrum, it is not possible to draw any definite conclusion about any PHEMA - PDMAM interaction.

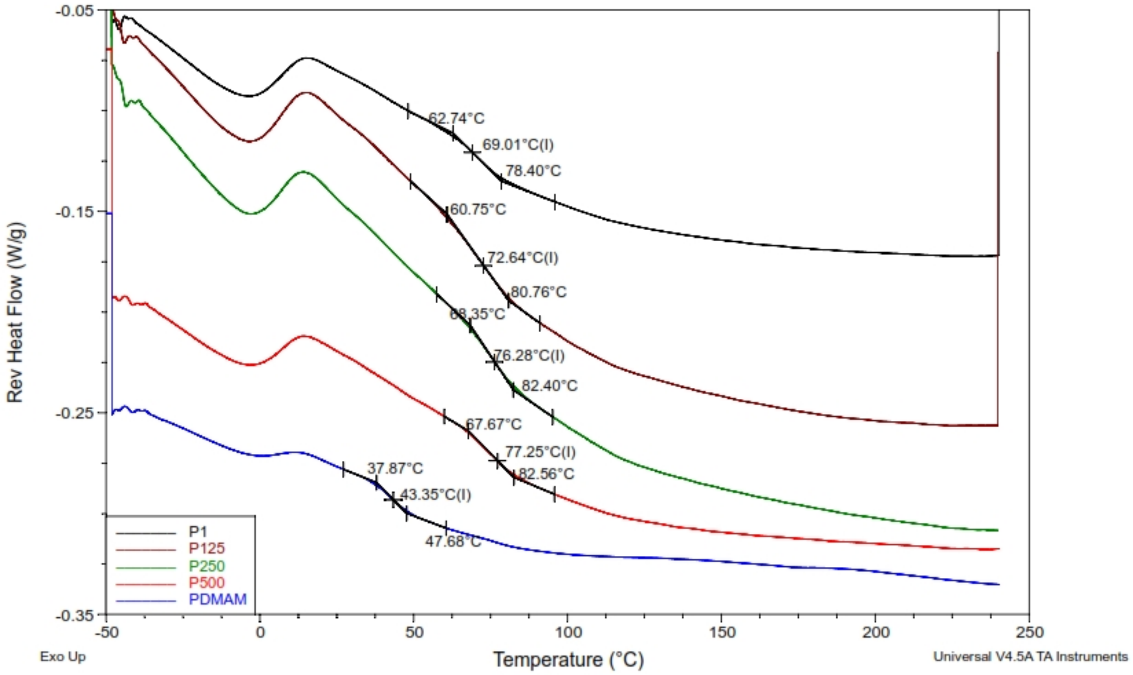

Figure S7. Reversing heat flow TMDSC thermograms of dry PHEMA and PDMAM SNs as well as for their IPNs

Table S2. Tg of non-loaded and DXP loaded PHEMA and PDMAM SNs as well as of their IPNs.

Sample	Tg [°C]	
	Non-loaded	*DXP loaded*
P1	69	80
P125	73	79
P250	76	77
P500	77	70
PDMAM	43	n.a.

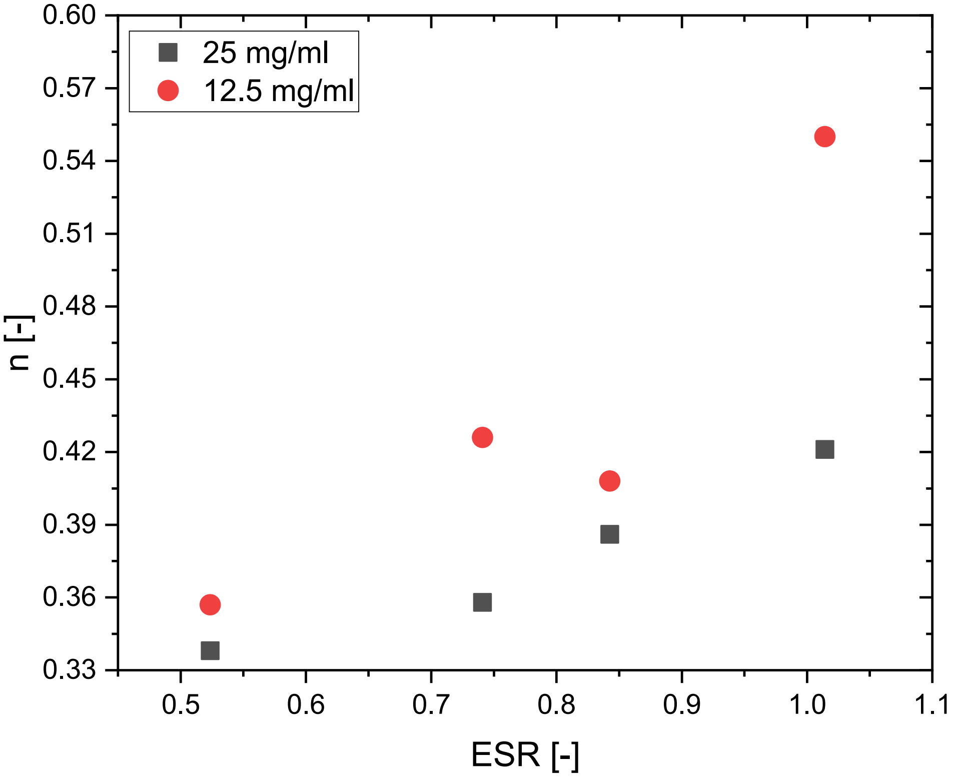

Figure S8. Correlation between diffusional exponents (n) of DXP release from PHEMA/PDMAM IPNs and their respective ESR.
